# Comparison of microbial molecular diagnosis efficiency within unstable template metagenomic DNA samples between qRT-PCR and chip-based digital PCR platforms

**DOI:** 10.5808/gi.23068

**Published:** 2023-12-29

**Authors:** Dongwan Kim, Junhyeon Jeon, Minseo Kim, Jinuk Jeong, Young Mok Heo, Dong-Geol Lee, Dong Keon Yon, Kyudong Han

**Affiliations:** 1 Department of Microbiology, College of Science & Technology, Dankook University, Cheonan 31116, Korea; 2 Department of Bioconvergence Engineering, Dankook University, Jukjeon 16890, Korea; 3 R&I Center, COSMAX BTI, Seongnam 13486, Korea; 4 Center for Digital Health, Medical Science Research Institute, Kyung Hee University Medical Center, Kyung Hee University College of Medicine, Seoul 02447, Korea; 5 Department of Pediatrics, Kyung Hee University College of Medicine, Seoul 02447, Korea; 6 HuNbiome Co., Ltd, R&D Center, Seoul 08503, Korea

**Keywords:** digital PCR, molecular diagnosis, quantitative real-time PCR, *Staphylococcus aureus*

## Abstract

Accurate and efficient microbial diagnosis is crucial for effective molecular diagnostics, especially in the field of human healthcare. The gold standard equipment widely employed for detecting specific microorganisms in molecular diagnosis is quantitative real-time polymerase chain reaction (qRT-PCR). However, its limitations in low metagenomic DNA yield samples necessitate exploring alternative approaches. Digital PCR, by quantifying the number of copies of the target sequence, provides absolute quantification results for the bacterial strain. In this study, we compared the diagnostic efficiency of qRT-PCR and digital PCR in detecting a particular bacterial strain (*Staphylococcus aureus*), focusing on skin-derived DNA samples. Experimentally, specific primer for *S. aureus* were designed at transcription elongation factor (*greA*) gene and the target amplicon were cloned and sequenced to validate efficiency of specificity to the *greA* gene of *S. aureus*. To quantify the absolute amount of microorganisms present on the skin, the variable region 5 (V5) of the 16S rRNA gene was used, and primers for *S. aureus* identification were used to relative their amount in the subject’s skin. The findings demonstrate the absolute convenience and efficiency of digital PCR in microbial diagnostics. We suggest that the high sensitivity and precise quantification provided by digital PCR could be a promising tool for detecting specific microorganisms, especially in skin-derived DNA samples with low metagenomic DNA yields, and that further research and implementation is needed to improve medical practice and diagnosis.

## Introduction

The development of the digital polymerase chain reaction (PCR) method, third-generation PCR equipment, has recently changed the research trend of the existing microbial molecular diagnosis field. Especially, a new digital PCR equipment ‘LOAA (Lab On An Array) digital real-time PCR analyzer (Optolane, Seongnam, Korea)’ has 1,000 times more fluorescence detection sensitivity than quantitative real-time polymerase chain reaction (qRT-PCR), second-generation PCR equipment, and is a high-performance PCR equipment that can detect each PCR amplification reaction in more than 20,000 nano-sized PCR reaction wells present on a single semiconductor chip-based micro electro mechanical system (MEMS) [1,2]. Above all things, unlike qRT-PCR, LOAA digital PCR is very useful in the field of specific pathogens or cancer diagnosis because it has independent ‘absolute gene quantitative analysis systems’ within equipment without a separate standard curve quantification analysis [3,4]. In the case of LOAA digital PCR, about 20,000 PCR reaction wells are distributed on a semiconductor chip, and PCR amplification and fluorescence detection proceeds from 0 or 1 template DNA molecule included per well. This characteristic valid a ‘Poisson distribution’ principle that can calculate positive and negative reactions on PCR amplification detected in each well in detail, enabling absolute quantities in the sample for a specific gene to be identified without a separate standard curve analysis. The formula for absolute quantification method within a sample of a specific gene based on the 'Poisson distribution' principle is as follows:


$$x=-\left(No.\;of\;total\;avaliable\;PCR\;well\right)\times log_{e}\;\left(\frac{No.\;of\;negative\;PCR\;well}{No.\;of\;total\;avaliable\;PCR\;well}\right)\;$$

However, although the digital PCR generates such high-efficiency molecular diagnostic outputs, the cost burden of one-time semiconductor-based experimental consumables is high, so if the quality and quality of template DNA are unstable during the experiment, consumer preference may decrease according to the experimental results. For example, metagenomic DNA (mDNA) samples isolated from skin applied in the human skin healthcare field are difficult to derive clear research results because their quantity and quality are often unstable due to various factors (e.g., Whether to wash, use skin care products, UV exposure, and individual living environment) affecting the growth environment of microorganisms on the human skin surface [5,6]. For this reason, the results of publishing research cases related to molecular microbial diagnosis using digital PCR in these unstable template DNA conditions, isolated from human skin, are insufficient, and for digital PCR to be optimized in various research fields in the future, it is necessary to identify these issues.

In this study, we aimed the comparing and verifying the quantitative efficiency of *Staphylococcus aureus* related to atopic disease occurrence within mDNA samples collected from lesions and non-lesions of seven atopic patients using qRT-PCR and digital PCR. We compared the specific gene detection and quantitative efficiency of the two PCR equipment under unstable template DNA conditions to evaluate the applicability of digital PCR to research fields such as skin microbiome studies that are highly affected by template DNA quality. In addition, by comparing the relative frequency difference of *S. aureus* between lesions and non-lesions skin sites in atopic patients, we demonstrated the reliability of the comparison results of quantitative efficiency for particular microorganisms between the two equipment [7]. Ultimately, we suggest through this study that digital PCR has high utilization value for various human healthcare industries related to molecular microbial diagnosis.  

## Methods and Results

### Particular bacterial species-specific primer and probe sets design method

In this study, we tried to verify the detection and quantification efficiency of digital PCR equipment for specific bacterial species within the unstable template mDNA sample. Therefore, for this verification, we selected *S. aureus*, which is expected to exist on skin sites samples of atopic patients, as a specific bacterial species considering previous studies showing that *S. aureus* affects the atopic disease [8-10]. First, we designed a *S. aureus*–specific primer and probe set to detect and quantify a particular bacterial species present within mDNA samples extracted from various microbial pools collected from the surface of human skin, and to compare the detection and quantification efficiency between qRT-PCR and digital PCR. The experimental verification process for the primer and probe set design we conducted is as follows.

#### Bacterial gene selection for targeting particular species

The *greA* gene encodes a transcription elongation factor that affects bacterial gene transcription by regulating gene promoters, thereby regulating the environmental adaptation of bacteria [11]. In addition, the *greA* gene is recognized as a bacterial housekeeping gene, which, like the bacterial 16S ribosomal RNA, is an evolutionarily conserved transcription factor widely distributed in prokaryotes [12].

#### Retrieval of coding sequence region base information from reference database

We obtained sequence information (FASTA format) of the *greA* gene coding sequence region (CDS) for 22 different *S. aureus* strains (at the strain level) annotated in the National Center for Biotechnology Information (NCBI) reference database (Supplementary Table 1). This process is essential to improve primer and probe binding accuracy and specificity, as the exact strain information of *S. aureus* present within the human skin-derived mDNA samples applied in this study is unclear.

#### Selection of target-specific sequence regions for PCR reaction

To select primer sequences that could detect all 22 different *S. aureus* strains, multiple sequence alignment method (MSA) of each CDS information was performed using BioEdit 7.2.5v software (Supplementary Fig. 1A). Two consistent regions identified through the MSA method were selected as forward and reverse primer sequences (Table 1).

#### In silico test for pre-validating primer binding specificity

We used the Oligo calc (http://biotools.nubic.northwetern.edu/) and ‘Oligo Analysis (http://www.operon.com/tools/oligo-analysis-tool.aspx)’ open web tools to pre-simulate the suitability of the selected primer pairs for experimental application (including Tm values, GC%, and probability of primer dimer formation). Next, the NCBI nucleotide Basic Local Alignment Search Tool (BLAST) tool was used to confirm species-specificity for each primer sequence and amplification region included in each primer pair (Supplementary Fig. 1B).

#### Specific probe design

Finally, we designed a specific probe sequence region within between each selected primer sequence (Table 1, Supplementary Fig. 1A). The fluorescent reporter dyes and quencher applied in the probe design were 6-FAM (6-carboxy fluorescein) and SFCQ1 (SFC probe, Cheongju, Korea), which were attached to the 5' and 3' end regions of the selected probe sequences, respectively.

### Comprehensive molecular genetical validation for primer/probe-specificity

To demonstrate the species-specificity of the pre-designed *S. aureus*–specific primers and probe through experimental validation, we set up a comparison group (Table 2). Information about the comparison group is as follows—positive control-1 (PC1): genomic DNA (gDNA) of a single strain of *S. aureus* ATCC6538; for gDNA extraction of PC, HiGene Genomic DNA Prep Kit For microorganisms (BIOFACT, Daejeon, Korea) was used, and all experimental procedures were performed according to the official protocol guide provided in the kit; negative control (NC) : ‘Siga-Microbial community DNA mix MBD0026’ contains genomic DNA from 10 bacterial species (*Akkermansia muciniphila*, *Bacillus subtilis*, *Burkholderia pyrrocinia*, *Escherichia coli*, *Enterococcus faecalis*, *Pseudomonas aeruginosa*, *Proteus mirabilis*, *Proteus vulgaris*, *Porphyromonas gingivalis*, and *Salmonella enterica*) are included in uniform proportions within one sample tube (Sigma-Aldrich, St. Louis, MO, USA); positive control-2 (PC2): genomic DNA of the *S. aureus* ATCC6538 was added to the NC; this control was set up to confirm that the specific-primers within the different microbial gDNA pools specifically bind to *S. aureus*. Therefore, we validated the experimental suitability (binding sensitivity) of *S. aureus*–specific primers and probes for application in qRT-PCR and digital PCR through a comprehensive molecular genetic experimental process. The experimental procedure was as follows.

#### General PCR validation for confirming primer binding specificity to *S. aureus*

**Fig. 1.**
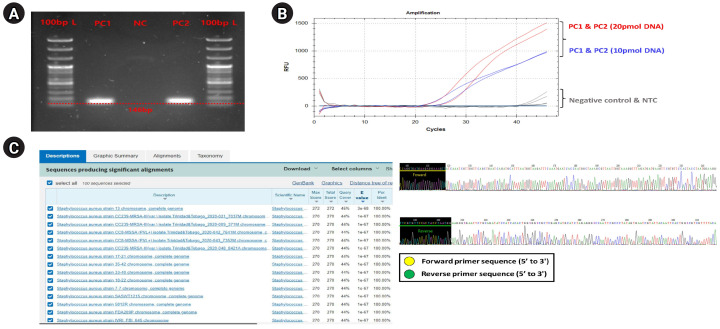
Comprehensive experimental validation for verifying designed *Staphylococcus aureus*–specific primer and probe set. Comprehensive validation results showing the experimental suitability evaluation of the designed *S. aureus*–specific primer and probe set for application to digital polymerase chain reaction (PCR) and quantitative real-time polymerase chain reaction (qRT-PCR) platforms. (A) Electrophoresis results reflected the PCR amplification for the *S. aureus* *greA* gene, verifying that specific binding was only on the *S. aureus* genomic DNA of the designed *S. aureus*–specific primer pair. (B) An amplification plot showing qRT-PCR results to establish optimal experimental conditions for applying primer and probe set designed for qRT-PCR and digital PCR platforms. (C) Sanger sequencing (right) and NCBI nucleotide BLAST test (left) results demonstrate that PCR amplification for *S. aureus* *greA* gene was successfully performed.

First, we performed a general PCR validation to confirm that our pre-designed primers specifically bind to the CDS region of the *greA* gene on the *S. aureus* genome (Fig. 1A). PCR verification confirmed the presence of a DNA amplicon band approximately 146 bp long in PC1 and PC2, and no amplicon DNA was identified in the NC. This shows that the primers we designed bind specifically to *S. aureus* and do not bind to the NC (T100 Thermal Cycler, Bio-Rad, Hercules, CA, USA). The running condition of the PCR is as follows: pre-denaturation 95.0°C, 5 min, denaturation 95.0°C, 30 s, annealing 59°C, 40 s, elongation 72.0°C, 15 s, final-extension 72.0°C, 30 s, and total PCR cycle was 30. Additionally, we confirmed that specific primers are bound normally to *S. aureus* genomic DNA within a complex microbial DNA pool through PCR reaction results of PC2 and could confirm the potential applicable for specific detection of *S. aureus* even within skin-derived mDNA samples to be applied in this study.

#### Optimizing the primer and probe experimental conditions for qRT-PCR and digital PCR

Next, to set the optimal experimental conditions of primers and probes for application to qRT-PCR and digital PCR, preferentially, we performed the qRT-PCR validation using PC1 template DNA sample with *S. aureus*–specific primer, and the primer concentration was set the 25 pmol, probe concentration was set 20 pmol, and 10 pmol conditions, respectively. The qRT-PCR (CFX Opus 96 Real-Time PCE System, Bio-Rad; BioFACT 2× Multi-Star Real-Time PCR Master Mix For Probe, UDG system) experimental conditions were as follows (Supplementary Table 2); pre-denaturation 95.0°C 10 min, denaturation 95.0°C 10 s, annealing/Flour detection 61°C 10 s, elongation 72.0°C 15 s, and total PCR cycle was 45). As a result, the resolution of detection fluorescence value (RFU; relative fluorescence units) for the *S. aureus* *greA* gene detected on the probe concentration condition of 20 pmol about PC1 and PC2 samples were found to be higher compared to the 10 pmol concentration condition, confirming that this experimental condition was the most optimal (Fig. 1B).

#### Standard curve analysis about positive control (*S. aureus*) using qRT-PCR

**Fig. 2.**
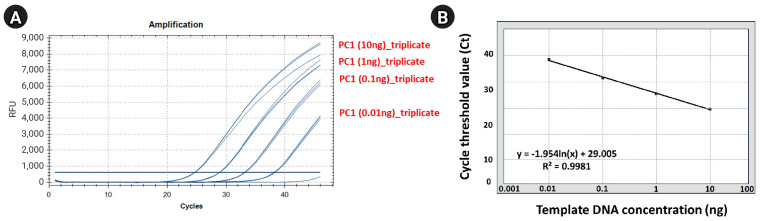
Standard curve calculation for positive control used to contrast the relative copy number of *Staphylococcus aureus* *greA* gene within the metagenomic DNA (mDNA) sample using the quantitative real-time polymerase chain reaction (qRT-PCR) method. These figures show that the standard curve for ‘positive control (PC1)’ was calculated according to the reflected Ct value per each serial-diluted DNA sample (10-fold serial dilution). (A) The qRT-PCR amplification plot reflecting the Ct value for each serial-diluted sample. (B) The standard curve graph was calculated based on the measured Ct value per each serial-diluted sample.

We performed a standard curve analysis using the Ct value reflected about the positive control to contrast the results of qRT-PCR–based detection and quantification of *S. aureus* within mDNA samples applied in this study (Fig. 2, Supplementary Table 3). We performed qRT-PCR in triplicate to confirm the reliability of the standard curve analysis results and performed a standard curve analysis using 10-fold serial-diluted template DNA (PC1) samples (10^1^, 10^0^, 10^-1^, and 10^-2^ diluted samples, respectively). Standard curve analysis for PC1 based on qRT-PCR showed that the average Ct values for each dilution factor were 10 ng, 24.67; 1 ng, 28.90; 0.1 ng: 33.20; 0.01 ng, 38.24; and the R2 value of trend line was calculated on the standard curve graph was approximately 0.99.

#### Primer binding specificity cross-validation through Sanger sequencing validation

Finally, we performed a Sanger sequencing (ABI 3500 Genetic Analyzer, Thermo Fisher Scientific, Waltham, MA, USA) and NCBI nucleotide BLAST test to verify that the primer designed in this study correctly targeted the *S. aureus* *greA* gene CDS region and used it for PCR amplification (Fig. 1C). In this verification step, since the PCR amplification region to be identified is too short and unsuitable for conducting the Sanger sequencing, we performed the sequencing process by expanding the gene reading region through the TA cloning method (TOPcloner TA Kit, Enzynomics, Daejeon, Korea [13]). Competent cells required in the transformation process for gene cloning were performed using DH5α Chemically Competent *E. coli* (Enzynomics), and white colonies (potentially successful transformation and *greA* gene ligation) identified in 37°C incubation were selectively harvested and applied for sequencing process. The primer pair applied for Sanger sequencing to read the *greA* gene amplicon region was the M13 region-specific primer set contained in the TA cloning plasmid vector sequence. As a result, we confirmed from the base-pair sequence reading data that both forward and reverse primer sequence information were included. And then, to verify that the generated sequencing data contained CDS regions (PCR amplicon region) of the *S. aureus* *greA* gene, we input the sequencing data into the NCBI nucleotide BLAST search engine and compared it with annotated bacterial classification information within the NCBI reference database. As a result of the BLAST test, we confirmed that only bacterial identification information for *S. aureus* was matched from the NCBI reference database. We could also confirm that the BLAST test detected various *S. aureus* strains (at the strain level) rather than a single bacterial strain. This result showed that the *S. aureus*–specific primer, considered up to the strain level, has broad detection efficiency for various *S. aureus* strains within any type of clinical sample.

### Detection and quantification for *S. aureus* using qRT-PCR method

We used human skin-derived mDNA samples as template DNA samples to confirm the efficiency of detection and quantification of specific microbial DNA within low molecule density template DNA using qRT-PCR. For this process, a total of 14 skin-derived clinical samples were obtained from seven patients with atopic dermatitis who visited the medical center (Kyung Hee University College of Medicine, Seoul, Republic of Korea), divided into lesion (case group) and non-lesion (control group) skin sites. Diagnosing atopic diseases of all participants was performed according to a dermatologist’s examination. Approval for the study protocol, informed consent forms, and related supporting documents was granted by the institutional review boards at the Korean Skin Research Center in South Korea (IRB No. HBABN01-220509-HRBR-E0113-01). All mDNA was extracted using the QIAamp PowerFecal Pro DNA kit (Qiagen, Hilden, Germany), and all experimental procedures were performed according to the official protocol guide provided with the kit. The average concentration values of extracted mDNA for each comparison group were checked with the control group 2.05 ng/μL and test group 4.10 ng/μL, respectively (Supplementary Table 4), by using a Thermo Scientific NanoDrop One/Onec Microvolume UV-Vis Spectrophotometer (Thermo Fisher Scientific). Next, we used qRT-PCR to determine the expected microbial frequency of *S. aureus* present within the 14 mDNA samples with a comparative analysis between groups to confirm the detection and quantification efficiency. The experimental procedure was as follows.

#### Normalization of bacterial quantification within each skin microbiome sample

**Fig. 3.**
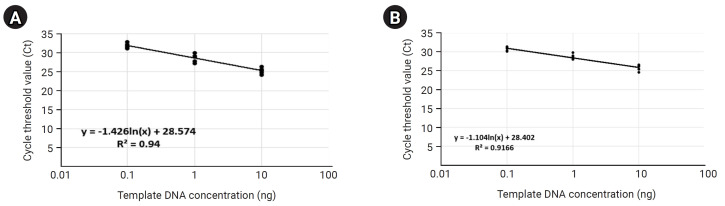
Quantitative real-time polymerase chain reaction (qRT-PCR)–based bacterial 16S V5 region standard curve analysis showing normalization of potential bacterial gDNA concentrations in each metagenomic DNA (mDNA) sample. The qRT-PCR–based standard graphs applying the bacterial 16S V5 variable region-specific primer show that the concentration of potential bacterial gDNA included within the mDNA to be used in this study has been normalized to almost the same (A, control group; B, case group).

Before the detection and quantitative analysis of *S. aureus* using qRT-PCR, we performed a qRT-PCR-based standard curve analysis using a primer pair targeting the V5 hyper-variable region included on the bacterial 16S ribosomal RNA (forward primer: 5′-GGATTAGATACCCTGGTA-3′, reverse primer: 5′-CCGTCAATTCMTTTRAGTTT-3′) to standardize the amount of potential bacterial DNA distributed within each 14 mDNA samples to equal condition (Table 3, Fig. 3). In this process, we normalized the concentration of all mDNAs to 10ng, and then we're going to confirm the uniformity of concentration and reliability of the experiment through standard curve graph analysis. We performed standard curve analysis about expected bacterial DNA quantity by diluting each normalized mDNA through a 10-fold serial dilution method (10^1^, 10^0^, 10^-1^, 10^-2^, respectively). The qRT-PCR experimental condition of standard curve analysis for 14 mDNA samples with bacterial 16S V5 primer pair was as follows: pre-denaturation 95.0°C 10 min, denaturation 95.0°C, 10 s, annealing/Flour detection 60°C, 15 s, elongation 72.0°C, 15 s, and total PCR cycle was 45. As a result, we confirmed that the average Ct values reflecting the potential bacterial DNA concentration for each dilution factor identified within the control group were 10 ng, 25.24; 1 ng, 28.67; 0.1 ng, 31.82; and the r^2^ value about the trend line calculated in the standard curve graph was 0.94. In the test group, we confirmed that the average Ct values for each diluted DNA sample were 10 ng, 25.72; 1 ng, 28.70; 0.1 ng, 30.81; and the r^2^ value was 0.9166. In the case of the ‘Test-7’ sample, considering that the concentration and quality of mDNA were low and unstable at 0.82 ng (A260/280: 2.37, A260/230: 0.01), we judged that it was difficult to quantify bacterial DNA by qRT-PCR because there were few potential bacterial communities present in this sample. Therefore, through this standard curve analysis, we could confirm that the potential bacterial DNA quantities within the 13 mDNA samples, except for the ‘Test-7’ sample, were equally normalized and suitable for detection and quantification analysis for *S. aureus*.

#### Evaluation of absolute and relative quantification effect for *S. aureus* via qRT-PCR

We evaluated the absolute and relative quantification effects of *S. aureus* in the skin microbiome sample through qRT-PCR using *S. aureus*–specific primer and probe set. Before the experiment, we confirmed that the distilled water used for making the qRT-PCR mixture solution was free of microbial contamination by checking the Ct value: N/A result reflected in the non-template DNA control (NTC). The qRT-PCR amplification conditions applied in this experiment were as follows: template DNA (mDNA) 10 ng; probe concentration 20 pmol, *GreA* primer concentration 25 pmol (this concentration condition was based on previous experimental results showing the most optimal running condition). As a result, we were not able to detect and quantify *S. aureus* within both comparison groups, which was not the expected result for this experiment (Table 4). Bacterial DNA normalization results confirmed by standard curve analysis demonstrated that the bacterial DNA in the skin microbiome sample was normalized to approximately the same amount, but due to the unstable quality of the mDNA (low concentration and low purity) and the low frequency of potential *S. aureus* expected to be present in it. Therefore, we judged that absolute and relative quantification of *S. aureus* within mDNA samples using the qRT-PCR method could not be meaningfully performed.

### Quantification of *S. aureus* within each skin microbiome sample using LOAA digital PCR

**Fig. 4.**
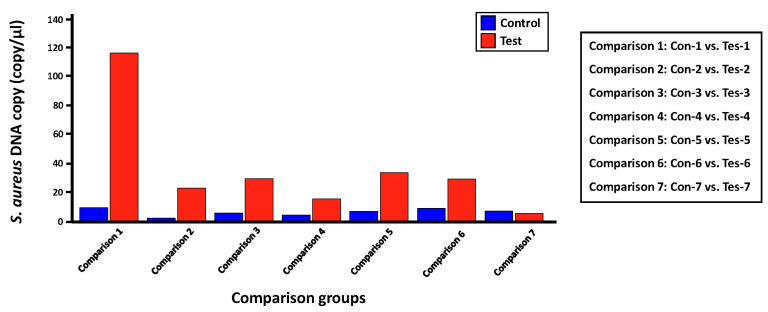
Bar graph showing the comparison of absolute quantitative values for *Staphylococcus aureus* *greA* gene measured within two comparison groups using digital polymerase chain reaction (PCR). This bar graph shows the difference in the expected gene copy number (= expected *S. aureus* bacterial frequency) of the *S. aureus* *greA* gene between each control (non-lesion site) and case group (lesion site).

We evaluated the applicability of the digital PCR platform to the microbial molecular diagnosis field by validating the detection and quantification effect for a proportion of *S. aureus*, which is expected to be present in each skin microbiome sample set in this study, using LOAA digital PCR equipment (Table 5, Fig. 4). The experimental group was set in the same as the process of detecting and quantifying *S. aureus* through the qRT-PCR method. Before conducting the digital PCR reaction for each comparison group, we confirmed that the number of expected copies of the *greA* gene within *S. aureus* genomic DNA in the PC1 and NC groups was 69,109 copies/µL and 0.00 copies/µL, respectively. Next, the average copies number of *S. aureus* *greA* gene identified in the control and test groups was 6.58 copies/µL and 36.28 copies/µL, respectively. However, in the case of the ‘Tes-7’ sample identified in the qRT-PCR results, it was confirmed that a low number of gene copies was detected, unlike the other mDNA samples in the test group, similar to the ‘16S V5 standard curve analysis’ result (Table 3), which was evaluated to have little bacterial genomic DNA density within this template DNA sample. Additionally, we could confirm that the potential bacterial frequency of *S. aureus* within the mDNA samples isolated from lesion skin sites of atopic patients was relatively high compared to the non-lesion skin sites. Considering previous studies showing that the dominant rate of *S. aureus* identified in atopic patients' lesions sites is higher than that of non-lesion sites, we could confirm the reliability of the *S. aureus* quantitative analysis results between each comparison group derived through LOAA digital PCR [14]. 

## Discussion

The digital PCR platform is spotlighted as third-generation PCR equipment that can absolutely quantify the copy number of a particular gene to be identified within the sample without calculating a separate standard curve [15]. However, due to the high-cost burden of consumables of digital PCR, consumers’ careful consideration of the quality of DNA samples or primer and probe suitability is required. Therefore, the present study evaluated the application possibility of microbial molecular diagnosis of digital PCR platform by confirming the effect of detection and quantification for specific microorganisms within mDNA samples in unstable conditions. First, we selected ‘*Staphylococcus aureus*’ as a particular microbe, and by designing the *S. aureus*–specific primer and probe set, we successfully evaluated its high experimental accuracy for targeted-species specificity through comprehensive molecular genetic experimental validation. Additionally, since we designed the primer pair considering the strain level of *S. aureus*, we suggest that the designed primer can detect a broad range of strain-level single strains present in various types of clinical samples. As a result of comparing the detection and quantification efficiency of *S. aureus* between qRT-PCR and digital PCR, unlike qRT-PCR, which could not confirm the relative proportion of *S. aureus* in any mDNA sample, we confirmed that the copy number of *S. aureus* *greA* gene was calculated in digital PCR. *S. aureus* inhabits human skin sites at a high proportion, with an average frequency of 20%–30% (approximately 50%–60% for atopic patients) [16]. However, we judged that detecting *S. aureus*, even with qRT-PCR with high fluorescence sensitivity, was challenging because the quality and quantity of the mDNA samples applied in this study were very low. Despite these conditions, we estimated that the semiconductor-based LOAA digital PCR equipment was capable of *S. aureus* *greA* gene amplification not detected by qRT-PCR because specific gene amplification is possible for each bacterial genomic DNA within each over 20,000 nano-size PCR well. Additionally, the applicability of digital PCR to microbial-related diagnosis and clinical research for particular diseases was confirmed by validating the relative frequency difference of *S. aureus* between atopic patients' lesions and non-lesions sites.

In summary, we evaluated in this study that digital PCR has a simpler experimental process spent quantifying and detecting specific genes compared to qRT-PCR and is significantly less time-consuming (qRT-PCR takes about 5 h, digital PCR takes about 1 h). Additionally, we verified that digital PCR has excellent performance on equipment in that it enables its independent absolute quantification application. Above all things, we could confirm that gene amplification, detection, and quantification are possible using digital PCR, even within DNA samples with unstable quality and quantity, such as skin-derived mDNA. However, digital PCR has high prices for semiconductor-based PCR response detection chips, limitations of not having more than three multiple fluorescent channel functionality support in equipment, and experimental inefficiency of ‘only one sample per equipment operation.’ However, considering the digital PCR's operating excellence in deriving high-quality output data, we suggest that digital PCR has high potential application value in the microbiome-based human healthcare field if it is used for cross-validation for quantification and detection for specific microorganisms to be identified.

## Figures and Tables

**Table 1.** Sequence information of *Staphylococcus aureus*–specific primer and probe sets

Primer/Probe name	Sequence (5' to 3')	Length (mer)	Tm (°C)	GC content (%)
*greA*_Forward	CCAGGTGATGAAGAGGAAAGTT	22	60.1	45
*greA*_Reverse	CCATTAGGTAGTGGAACACGAA	22	60.1	45
*greA*_Probe	56-FAM/TCAAATCGTTGGTTCAGCTGAATCAGAT/3SFCQ1	28	65.6	39

**Table 2.** Overall samples information of samples applied to this study

Comparison type	Bacterial taxonomy	Comparison type	Sample	Sample type	Clinical type
Positive control-1 (PC1)	*Staphylococcus aureus *ATCC6538	Control group	Con-1	Human skin	Healthy sites
Negative control (NC)	*Akkermansia muciniphila*		Con-2		
	*Bacillus subtilis*		Con-3		
	*Burkholderia pyrrocinia*		Con-4		
	*Escherichia coli*		Con-5		
	*Enterococcus faecalis*		Con-6		
	*Pseudomonas aeruginosa*		Con-7		
	*Proteus mirabilis*	Test group	Tes-1	Human skin	Lesion sites
	*Proteus vulgaris*		Tes-2		
	*Porphyromonas gingivalis*		Tes-3		
	*Salmonella enterica*		Tes-4		
Positive control-2 (PC2)	*Staphylococcus aureus *ATCC6538		Tes-5		
	*Akkermansia muciniphila*		Tes-6		
	*Bacillus subtilis*		Tes-7		
	*Burkholderia pyrrocinia*				
	*Escherichia coli*				
	*Enterococcus faecalis*				
	*Pseudomonas aeruginosa*				
	*Proteus mirabilis*				
	*Proteus vulgaris*				
	*Porphyromonas gingivalis*				
	*Salmonella enterica*				
					

**Table 3.** Standard curve calculation results of each 10-fold diluted sample using qRT-PCR assay

Sample	10 ng_Ct value	1 ng_Ct value	0.1 ng_Ct value	Target gene
Con-1	25.23	28.83	31.91	Bacterial 16S rRNA V5 region
Con-2	26.34	29.85	32.72	
Con-3	25.35	29.04	31.40	
Con-4	24.12	27.77	31.01	
Con-5	25.40	28.92	32.05	
Con-6	25.54	29.08	32.03	
Con-7	24.72	27.18	31.55	
NTC	38.37	N/A	N/A	
Con-Avg	25.24	28.67	31.82	-
Tes-1	26.52	28.03	31.14	Bacterial 16S rRNA V5 region
Tes-2	26.19	28.52	30.94	
Tes-3	25.40	28.97	30.08	
Tes-4	24.53	28.03	30.30	
Tes-5	25.43	29.84	31.38	
Tes-6	26.21	28.81	30.94	
Tes-7	N/A	N/A	N/A	
NTC	N/A	N/A	N/A	
Tes-Avg	25.72	28.70	30.81	-

qRT-PCR, quantitative real-time polymerase chain reaction; Ct, cycle threshold; NTC, non-template DNA control; Avg, average Ct value; N/A, not applicable.

**Table 4.** Quantification of *Staphylococcus aureus* using qRT-PCR method of each comparison group

Sample	10 ng/μL Ct value	Target gene	Fluorescence
Con-1	43.81	*S. aureus greA*	FAM
Con-2	43.92		
Con-3	45.91		
Con-4	43.32		
Con-5	44.74		
Con-6	43.73		
Con-7	43.75		
NTC	N/A		
Tes-1	N/A	*S. aureus greA*	FAM
Tes-2	N/A		
Tes-3	N/A		
Tes-4	N/A		
Tes-5	N/A		
Tes-6	N/A		
Tes-7	N/A		
NTC	N/A		

qRT-PCR, quantitative real-time polymerase chain reaction; Ct value, cycle threshold value; NTC, non-template DNA control; N/A, not applicable.

**Table 5.** Absolute quantification of *Staphylococcus aureus* copy number within human skin microbiome using digital PCR

Samples	Quantification (DNA copy/μL)	Expected total *S. aureus* copy number (10 μL sample)
Con-1	9.67	97
Con-2	2.47	25
Con-3	5.84	58
Con-4	4.53	45
Con-5	7.12	71
Con-6	9.20	92
Con-7	7.26	73
Tes-1	115.98	1,160
Tes-2	23.04	230
Tes-3	29.63	296
Tes-4	15.69	157
Tes-5	33.80	338
Tes-6	29.39	294
Tes-7	5.74	57
PC1	69,109	691,091
NC	0	0

PCR, polymerase chain reaction; PC1, positive control 1; NC, negative control.

## Supplementary Materials

Supplementary Table. 1. NCBI database annotation information about greA gene of each *Staphylococcus aureus* strains

Supplementary Table 2. Comparison of Ct value according to each probe amount for optimizing the detection efficiency of *Staphylococcus aureus*

Supplementary Table 3. Standard curve analysis about positive control 1 (PC1)

Supplementary Table 4. Quantity and quality check results for each mDNA sample

Supplementary Fig. 1. Primer/probe set design process for detection and quantification of relative bacterial load for Staphylococcus aureus. These figures show the design process of the primer/probe set for quantitative real-time polymerase chain reaction to detect and quantify the relative bacterial load of Staphylococcus aureus. (A) Multiple sequence alignment results using the Bioedit 7.2 ver. bioinformatics tool show that the primer/probe was designed within consistent regions (highlighted by green box) on the greA gene of all S. aureus strains (strain level) annotated on the NCBI database (greA_F: forward primer sequence region; greA_R: reverse primer sequence region; greA_P1: probe sequence region). (B) Basic Local Alignment Search Tool (BLAST) test results showing the species-specificity for S. aureus of primer set.

## References

[1] Chen B, Jiang Y, Cao X, Liu C, Zhang N, Shi D (2021). Droplet digital PCR as an emerging tool in detecting pathogens nucleic acids in infectious diseases. Clin Chim Acta.

[2] Khan MS, Tariq MO, Nawaz M, Ahmed J (2021). MEMS sensors for diagnostics and treatment in the fight against COVID-19 and other pandemics. IEEE Access.

[3] Lambrescu I, Popa A, Manole E, Ceafalan LC, Gaina G (2022). Application of droplet digital PCR technology in muscular dystrophies research. Int J Mol Sci.

[4] Lee SS, Park JH, Bae YK (2021). Comparison of two digital PCR methods for EGFR DNA and SARS-CoV-2 RNA quantification. Clin Chim Acta.

[5] Castelino M, Eyre S, Moat J, Fox G, Martin P, Ho P (2017). Optimisation of methods for bacterial skin microbiome investigation: primer selection and comparison of the 454 versus MiSeq platform. BMC Microbiol.

[6] Lee H, Jeong J, Oh Y, Lee CJ, Mun S, Lee DG (2021). Comparative analysis of human facial skin microbiome between topical sites compared to entire face. Genes Genomics.

[7] Vickery TW, Ramakrishnan VR, Suh JD (2019). The role of *Staphylococcus aureus* in patients with chronic sinusitis and nasal polyposis. Curr Allergy Asthma Rep.

[8] Di Domenico EG, Cavallo I, Capitanio B, Ascenzioni F, Pimpinelli F, Morrone A (2019). *Staphylococcus aureus* and the cutaneous microbiota biofilms in the pathogenesis of atopic dermatitis. Microorganisms.

[9] Krishna S, Miller LS (2012). Host-pathogen interactions between the skin and *Staphylococcus aureus*. Curr Opin Microbiol.

[10] Parlet CP, Brown MM, Horswill AR (2019). Commensal staphylococci influence *Staphylococcus aureus* skin colonization and disease. Trends Microbiol.

[11] Suo B, Guan P, Dong Z, Zeng Y, Fan S, Fan H (2022). Comparative transcriptomic analysis of *Staphylococcus aureus* reveals the genes involved in survival at low temperature. Foods.

[12] Cui G, Wang J, Qi X, Su J (2018). Transcription elongation factor GreA plays a key role in cellular invasion and virulence of Francisella tularensis subsp. novicida. Sci Rep.

[13] Crossley BM, Bai J, Glaser A, Maes R, Porter E, Killian ML (2020). Guidelines for Sanger sequencing and molecular assay monitoring. J Vet Diagn Invest.

[14] Ogonowska P, Gilaberte Y, Baranska-Rybak W, Nakonieczna J (2020). Colonization with *Staphylococcus aureus* in atopic dermatitis patients: attempts to reveal the unknown. Front Microbiol.

[15] Devonshire AS, Honeyborne I, Gutteridge A, Whale AS, Nixon G, Wilson P (2015). Highly reproducible absolute quantification of Mycobacterium tuberculosis complex by digital PCR. Anal Chem.

[16] Kwon S, Choi JY, Shin JW, Huh CH, Park KC, Du MH (2019). Changes in lesional and non-lesional skin microbiome during treatment of atopic dermatitis. Acta Derm Venereol.

