# Supplementary material for: Comparison of microbial molecular diagnosis efficiency within unstable template metagenomic DNA samples between qRT-PCR and chip-based digital PCR platforms
Source: Genomics Inform. 2023 Dec 29;21(4):e52. doi: 10.5808/gi.23068 (PMC10788361; doi:10.5808/gi.23068)
Supplement: Supplementary Table. 1. — NCBI database annotation information about greA gene of each Staphylococcus aureus strains [file gi-23068-Supplementary-Table-1.pdf]

Supplementary Table 1. NCBI database annotation information about *greA* gene of each *Staphylococcus aureus* strains

No.	Genome accession No.	Protein accession No.	Gene name	Bacterial taxonomy	Strain No.
1	CP065857.1	QPV65454.1	*GreA*	*Staphylococcus aureus*	CC1153-MRSA
2	CP032161.1	QOE19269.1	*GreA*	*Staphylococcus aureus*	SA H27
3	CP053101.1	QJR42020.1	*GreA*	*Staphylococcus aureus*	EDCC5398
4	CP033117.1	QFZ04969.1	*GreA*	*Staphylococcus aureus*	Zhenghai
5	CP035101.1	QCY30254.1	*GreA*	*Staphylococcus aureus*	ATCC 12600
6	CP039156.1	QBZ85874.1	*GreA*	*Staphylococcus aureus*	WCUH29
7	CP035670.1	QBB16477.1	*GreA*	*Staphylococcus aureus*	VB9352
8	CP034349.1	AZL91607.1	*GreA*	*Staphylococcus aureus*	80wphwpl
9	CP009681.1	AIU84634.1	*GreA*	*Staphylococcus aureus*	Gv69
10	FFZR01000002.1	CXK22744.1	*GreA*	*Staphylococcus aureus*	st502
11	FGBJ01000004.1	CXL59646.1	*GreA*	*Staphylococcus aureus*	st676
12	FHAD01000001.1	CYC13506.1	*GreA*	*Staphylococcus aureus*	st3125
13	CSIK01000003.1	CPD51867.1	*GreA*	*Staphylococcus aureus*	USFL308
14	CSBH01000002.1	COW80319.1	*GreA*	*Staphylococcus aureus*	USFL206
15	CABFMG010000020.1	VTY30692.1	*GreA*	*Staphylococcus aureus*	BgEED30
16	CP003045.1	AFH69850.1	*GreA*	*Staphylococcus aureus*	71193
17	AIDT01000001.1	EIA15394.1	*GreA*	*Staphylococcus aureus*	DR10
18	AP017922.1	BBA24254.1	*GreA*	*Staphylococcus aureus*	JP080
19	CP054876.1	QKV60182.1	*GreA*	*Staphylococcus aureus*	Dresden-275757
20	CP062279.1	QOJ76193.1	*GreA*	*Staphylococcus aureus*	MVF-7
21	CP032160.1	QOE85244.1	*GreA*	*Staphylococcus aureus*	SA G5
22	CP032468.1	QOE21758.1	*GreA*	*Staphylococcus aureus*	SA H29
