# Supplementary material for: Comparison of microbial molecular diagnosis efficiency within unstable template metagenomic DNA samples between qRT-PCR and chip-based digital PCR platforms
Source: Genomics Inform. 2023 Dec 29;21(4):e52. doi: 10.5808/gi.23068 (PMC10788361; doi:10.5808/gi.23068)
Supplement: Supplementary Table 2. — Comparison of Ct value according to each probe amount for optimizing the detection efficiency of Staphylococcus aureus [file gi-23068-Supplementary-Table-2.pdf]

**Supplementary Table 2.** Comparison of Ct value according to each probe amount for optimizing the detection efficiency of *Staphylococcus aureus*

| Sample | Probe amount | Fluorescence channel | Target gene | Ct value |
|---|---|---|---|---|
| PC1 | 20 pmol | FAM (green) | *S. aureus greA* | 21.29 |
| PC2 | | | | 22.93 |
| NC | | | | 40.61 |
| NTC | | | | 40.51 |
| PC1 | 10 pmol | | | 20.41 |
| PC2 | | | | 21.79 |
| NC | | | | 38.53 |
| NTC | | | | 39.88 |

Ct value, cycle threshold; PC, positive control; NC, negative control; NTC, non-template DNA control.
