# Supplementary material for: Comparison of microbial molecular diagnosis efficiency within unstable template metagenomic DNA samples between qRT-PCR and chip-based digital PCR platforms
Source: Genomics Inform. 2023 Dec 29;21(4):e52. doi: 10.5808/gi.23068 (PMC10788361; doi:10.5808/gi.23068)
Supplement: Supplementary Table 3. — Standard curve analysis about positive control 1 (PC1) [file gi-23068-Supplementary-Table-3.pdf]

Supplementary Table 3. Standard curve analysis about positive control 1 (PC1)

Biological replication	Ct value	Quantity (ng)	Ct value	Quantity (ng)	Ct value	Quantity (ng)	0.01 ng	Quantity (ng)	NTC
1	24.72	10	28.96	1	33.28	0.1	38.35	0.01	N/A
2	24.62		28.86		33.17		38.19		
3	24.68		28.89		33.16		38.18		
Average value	24.67	-	28.90	-	33.20	-	38.24	-	N/A

Ct, cycle threshold; NTC, non-template DNA control; N/A, not applicable.
