# Supplementary material for: Comparison of microbial molecular diagnosis efficiency within unstable template metagenomic DNA samples between qRT-PCR and chip-based digital PCR platforms
Source: Genomics Inform. 2023 Dec 29;21(4):e52. doi: 10.5808/gi.23068 (PMC10788361; doi:10.5808/gi.23068)
Supplement: Supplementary Table 4. — Quantity and quality check results for each mDNA sample [file gi-23068-Supplementary-Table-4.pdf]

Supplementary Table 4. Quantity and quality check results for each mDNA sample

Sample	Nucleic acid (ng/μL)	A260/A280	A260/A230
Con-1	2.77	1.06	0.03
Con-2	1.69	1.13	0.02
Con-3	1.58	1.09	0.01
Con-4	1.49	0.88	0.04
Con-5	2.19	1.08	0.02
Con-6	2.73	1.16	0.02
Con-7	1.91	0.93	0.02
Tes-1	6.47	1.37	0.02
Tes-2	3.98	1.16	0.01
Tes-3	5.83	1.05	0.02
Tes-4	2.41	0.93	0.01
Tes-5	3.60	1.16	0.01
Tes-6	3.61	1.17	0.02
Tes-7	0.82	2.37	0.01

mDNA, metagenomic DNA.
