# Supplementary material for: Comparison of microbial molecular diagnosis efficiency within unstable template metagenomic DNA samples between qRT-PCR and chip-based digital PCR platforms
Source: Genomics Inform. 2023 Dec 29;21(4):e52. doi: 10.5808/gi.23068 (PMC10788361; doi:10.5808/gi.23068)
Supplement: Supplementary Fig. 1. — Primer/probe set design process for detection and quantification of relative bacterial load for Staphylococcus aureus. These figures show the design process of the primer/probe set for quantitative real-time polymerase chain reaction to detect and quantify the relative bacterial load of Staphylococcus aureus. (A) Multiple sequence alignment results using the Bioedit 7.2 ver. bioinformatics tool show that the primer/probe was designed within consistent regions (highlighted by green box) on the greA gene of all S. aureus strains (strain level) annotated on the NCBI database (greA_F: forward primer sequence region; greA_R: reverse primer sequence region; greA_P1: probe sequence region). (B) Basic Local Alignment Search Tool (BLAST) test results showing the species-specificity for S. aureus of primer set. [file gi-23068-Supplementary-Fig-1.pdf]

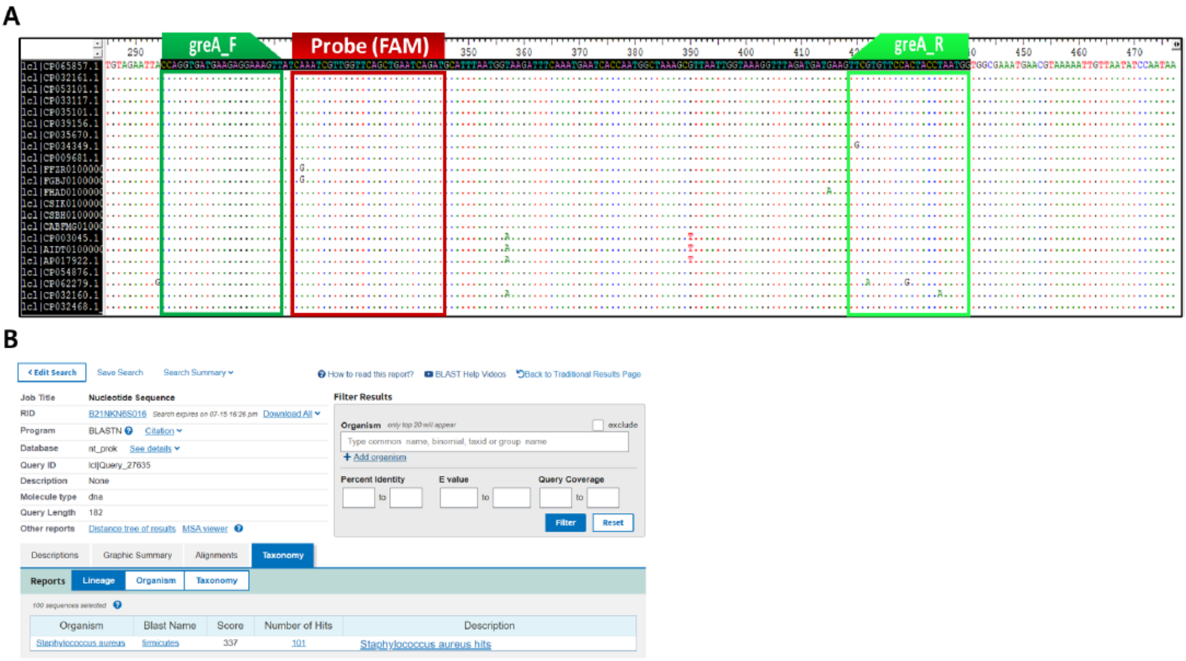

Supplementary Fig. 1.
